# Provider Bias in prescribing opioid analgesics: a study of electronic medical Records at a Hospital Emergency Department

**DOI:** 10.1186/s12889-021-11551-9

**Published:** 2021-08-06

**Authors:** Lisa A. Keister, Chad Stecher, Brian Aronson, William McConnell, Joshua Hustedt, James W. Moody

**Affiliations:** 1 Department of Sociology, Duke Network Analysis, Sanford School of Public Policy, Duke University, Durham, NC 27705 USA; 2 College of Health Solutions, Arizona State University, Phoenix, AZ 85004 USA; 3 The Adecco Group, 10151 Deerwood Park Blvd bldg 200 ste 101, Jacksonville, FL 32256 USA; 4 Department of Sociology, Florida Atlantic University, 777 Glades Road | CU 97 Rm 253, Boca Raton, FL 33431 USA; 5 Department of Orthopedics, University of Arizona College of Medicine-Phoenix, Phoenix, AZ 85004 USA; 6 Department of Sociology, Duke Network Analysis, Duke University, Durham, NC 27705 USA

**Keywords:** Opioids, Prescription bias, Emergency departments, Electronic medical records, Undertreatment, Crowding, Inequality

## Abstract

**Background:**

Physicians do not prescribe opioid analgesics for pain treatment equally across groups, and such disparities may pose significant public health concerns. Although research suggests that institutional constraints and cultural stereotypes influence doctors’ treatment of pain, prior quantitative evidence is mixed. The objective of this secondary analysis is therefore to clarify which institutional constraints and patient demographics bias provider prescribing of opioid analgesics.

**Methods:**

We used electronic medical record data from an emergency department of a large U.S hospital during years 2008–2014. We ran multi-level logistic regression models to estimate factors associated with providing an opioid prescription during a given visit while controlling for ICD-9 diagnosis codes and between-patient heterogeneity.

**Results:**

A total of 180,829 patient visits for 63,513 unique patients were recorded during the period of analysis. Overall, providers were significantly less likely to prescribe opioids to the same individual patient when the visit occurred during higher rates of emergency department crowding, later times of day, earlier in the week, later years in our sample, and when the patient had received fewer previous opioid prescriptions. Across all patients, providers were significantly more likely to prescribe opioids to patients who were middle-aged, white, and married. We found no bias towards women and no interaction effects between race and crowding or between race and sex.

**Conclusions:**

Providers tend to prescribe fewer opioids during constrained diagnostic situations and undertreat pain for patients from high-risk and marginalized demographic groups. Potential harms resulting from previous treatment decisions may accumulate by informing future treatment decisions.

**Supplementary Information:**

The online version contains supplementary material available at 10.1186/s12889-021-11551-9.

## Background

Physicians do not prescribe opioid analgesics for pain treatment equally across groups. Latino and African Americans, for example, are significantly less likely than whites to receive opioid analgesics following a major surgical procedure [1–4], and opioid prescriptions rates for women and senior citizens appear low relative to their reports of chronic pain [4–6]. Such disparities in pain treatment may pose significant public health concerns. Many recognize pain as the “fifth vital sign” of health [7, 8]; undertreatment of pain is associated with a variety of disadvantages such as diminished quality of life, physical functioning, mental acuity, sexual functioning, and sleep. Undertreated pain is also associated with increased (and costly) returns to the hospital [9, 10]. Therefore, it is critical to identify factors that might bias doctors’ treatment of pain.

Undertreatment for pain results from many factors that operate in combination including pain’s ambiguous diagnostic criteria, cultural stereotypes around particular groups’ experience of pain, and institutional constraints that affect doctors’ ability to diagnose pain. Because pain often lacks clear physical markers, doctors are forced to elicit information directly from patients to determine whether opioid analgesic treatment is necessary [11–13]. However, subjective assessments of patients’ pain needs are highly subject to implicit bias [14]. For example, doctors often mistakenly assume that African Americans have more pain tolerance and have more illicit motivations for seeking pain treatment than do whites [15–19]. Long-standing stereotypes about women’s lack of pain tolerance appear to bias doctors to both over-treat and undertreat women’s pain [4, 20]. Preferences for patients who are culturally similar to doctors may lead doctors to reduce attention to the suffering of patients from lower socio-economic groups [11, 16, 21, 22] thereby limiting doctors’ abilities to make informed diagnoses. Institutional constraints also play a role in doctors’ treatment decisions. Some evidence suggests that doctors are more likely to over-treat pain during periods of increased time-pressure, such as when it is late at night or when emergency departments are crowded [15, 23]. Such time constraints may intensify doctors’ implicit biases by increasing their likelihood to rely on implicit assumptions about patient diagnostic categories to inform treatment [13, 24].

Although there are many studies about how institutional constraints and cultural stereotypes influence doctors’ treatment of pain, quantitative evidence is mixed. Recent research suggests that doctors are biased towards over-treating patients during periods of time constraint and hospital crowding [15, 23], but such claims seem to contradict a small body of evidence that doctors are averse to prescribing opiates when under time-constraint [4, 25]. Gendered biases in pain treatment have been documented in a fairly large body of ethnographic research [4, 20], but these biases are not well-supported by quantitative evidence [3, 26, 27]. In fact, even though race may be the best documented source of provider bias in opioid pain treatment, prior studies have only examined these biases in reference to Latino and African American patients. There have been no substantial attempts to explain how other racial backgrounds, such as Asian heritage, is associated with pain treatment.

Sociological research suggests that some discrepant findings may be due to overlooked mediators and moderators of other cultural and institutional constraints [14, 15, 20, 28]. For example, high frequency of opioid treatment of pain for patients late at night may reflect selection biases in patients’ medical histories rather than doctors’ time pressures. Many demographic correlates with pain treatment might be induced by related factors, such as patient race, gender, age, marital status, or medical history. Ignoring how multiple factors simultaneously contribute to bias in pain treatment likely subjects much prior research to omitted variable bias.

To understand how institutional constraints and patient demographics influence pain treatment, this paper examines a large longitudinal dataset of electronic medical records gathered at an emergency department of a large private hospital in the United States. We study how opioid treatment for pain is simultaneously influenced by multiple factors and how this may bias pain treatment. Specifically, we examine how pain treatment is influenced by the intersection of emergency department crowding and race, the time patients visited the emergency department, patients’ medical histories, patients’ diagnoses, age, sex, marital status, and year of analysis. This is one of the largest and most comprehensive longitudinal studies of demographic and institutional predicates of opioid pain treatment in the literature, and it is one of only a few studies that examine multiple factors associated with pain treatment in tandem and over time. Our findings inform dominant hypotheses about provider bias, clarify prior mixed evidence, and provide evidence for under-explored avenues of bias in pain treatment.

## Methods

We assess provider biases in pain treatment with data from an electronic medical record (EMR) system of a large university hospital in the United States. We focus our analysis on EMR data collected from 2008 to 2014 at the hospital’s emergency department (ED) (*n* = 180,829 patient visits; 63,513 unique individuals). Roughly 54.1% (34,361) patients are seen only once in the ED, while 18.6% (11,844) visit the ED twice, 8.9% (5669) visit the ED three times, 5.0% (3180) visit the ED four times, and 67.4% (8459) visit the ED five or more times over the sample period. Consistent with prior work on doctor biases in pain treatment, we focus on EDs because the majority of patients who visit EDs (prior estimates as high as 70% [29, 30]) do so for pain treatment. EDs are also heavily subject to crowding and often serve patients who come from a wide array of socioeconomic backgrounds – both factors that are critical to our analysis. Ethics oversight was provided by the Duke University Institutional Review Board.

### Statistical analyses

To determine factors associated with provider bias in opioid pain treatment, we estimate a series of three generalized linear mixed models with a logit link function that predict whether a patient was prescribed opioids during a given ED visit based on a variety of contextual and demographic variables. Our models are ordered by complexity. Our first model includes patient-level random intercepts to account for repeated observations for individual patients. Our second model includes both patient-level random intercepts and fixed effects for each of 484 common International Classification of Diseases [31] (ICD-9) diagnoses recorded during the ED visit to account for differences in patients’ medical needs. Controlling for patient diagnoses should prevent spurious associations between opioid treatment and patients’ characteristics as a result of the association of particular medical conditions with demographic backgrounds. Our final model controls for ICD-9 diagnoses and estimates patient-level fixed-effects rather than random intercepts. Including patient-level fixed effects allows us to closely track how changes in the medical environment affect providers’ likelihood to prescribe opioid analgesics to individual patients while adjusting for (observed and unobserved) time-invariant patient characteristics. All analyses were conducted using the glmer package in R version 3.6.3.

### Measures

The dependent variable in all models is whether individuals were *prescribed any opioids during the patient visit*. Because our data include detailed patient records, we are able to observe and measure whether the patient received a prescription. This is a binary measure that tracks whether a patient received at least one of the following generic opioid prescriptions (listed in order of frequency): oxycodone, morphine, hydromorphone, fentanyl, hydrocodone, codeine, methadone, tapentadol, or meperidine.^1^

We use five variables to measure ED context. First, we measure *ED crowding*, our most central contextual measure, as the number of patients who visited in the last 4 hours. This variable accounts for the degree to which providers are under time pressure. We chose 4 hours as our cutoff in order to leverage available data and account for the fact that many EDs set four-hours as their target maximum wait time [32]. Second, we test whether ED crowding is more strongly associated with prescribing opioids to patients based on patient race by including an interaction between *ED crowding* and respondent race *(ED crowding x black).* Third, we include several indicators for time of day to adjust for different patient counts throughout the day: 12 am-5:59 am (*overnight*, reference), 6 am-11:59 am (*morning*), 12 pm-5:59 pm (*afternoon*), and 6 pm-11:59 pm (*evening*). Fourth, we include an indicator that the ED visit occurred on a *weekend* (Saturday or Sunday) to control for lower patient numbers during the weekend. Finally, we include an indicator for *year* of visit from 2008 (reference) to 2014. The year variable accounts for a cautioning effect of the opioid crisis on prescribing opioids. After controlling for routine variations in ED visits based on time of day, week, and year, the *ED crowding* variable captures the quasi-random fluctuation in ED visits that are unexpected and not likely to be correlated with patient characteristics or other patient-level determinants of physicians’ opioid prescribing.

We also control for demographic factors that are known to be associated with opioid prescriptions. We include measures of patient *age* coded as 10-year age indicator variables (age 19 and under [reference], age 20–29, 30–39, etc.) to test whether providers are more hesitant to prescribe opioid analgesics to younger or older citizens. We measure race using indicator variables for *white* (reference), *black*, *Latino*, *Asian*, and *other* race to test for racial biases against prescribing opioids to people of color. We include an indicator for *sex* (reference = *female*) and we include four *race-sex* interaction terms to test whether women or women of color have distinct disadvantages when seeking pain treatment. We include indicators for marital status (*unmarried* [reference], *married*, *divorced*, *widowed*, and *separated*) to test whether doctors prescribe opioid analgesics more often to individuals who have familial ties than to individuals who are single. Finally, we account for patient medical histories by modeling the number of times patients had received any opioid prescriptions during prior ED visits (*previously prescribed number*). Because our coverage of patients’ medical histories increases over time, we also include year interactions with patient prescription counts and exclude the first year of our available data (2007) from analysis.

## Results

Table 1 presents descriptive statistics for all variables based on all EMR events at one hospital emergency department. Consistent with prior estimates of opioid prescriptions at EDs, one-third of patient visits to the ED resulted in an opioid prescription. The majority of patient visits were by individuals who were female (57%), black (60%0) and unmarried (53%). These numbers partially reflect a greater propensity for female and black individuals to visit EDs than males or whites and partially reflect the demographic makeup of the county (excluded for confidentiality).

**Table 1:** Descriptive Statistics for all EMR Events at a Hospital Emergency Department, 2008–2014 (*n* = 180,829)

	(Percent)	Number of EMR Events
**Dependent Variable**		
Prescribed opioid^a^	(33.00)	59,717
**Contextual Variables**		
ED crowding^b^ (mean; SD)	13.92	6.08
Time of day		
*12 am-5:59 am (ref.)*	(11.66)	21,070
*6 am-11:59 am*	(29.40)	53,166
*12 pm-5:59 pm*	(33.32)	60,257
*6 pm-11:59 pm*	(25.62)	46,336
Weekend	(26.43)	47,800
Year		
*2008*	(14.20)	25,689
*2009*	(15.29)	27,651
*2010*	(14.27)	25,808
*2011*	(14.89)	26,919
*2012*	(14.37)	25,990
*2013*	(13.24)	23,945
*2014*	(13.73)	24,827
Prev. prescribed (#)^c^ (median; IQR)	1	3
**Demographic Variables**		
Female	(56.93)	102,952
Age (mean; SD)	44.81	16.85
Race		
*White (ref.)*	(28.72)	51,925
*Black*	(59.77)	108,088
*Latino*	(7.25)	13,108
*Asian*	(1.00)	1819
*Other*	(3.26)	5889
Marital Status		
*Unmarried (ref.)*	(53.28)	96,340
*Married*	(26.74)	48,345
*Divorced*	(8.97)	16,236
*Widowed*	(7.01)	12,667
*Separated*	(4.00)	7241

Sample includes all EMR events representing 63,513 unique individuals; ^a^ Whether visit resulted in an opioid prescription. ^b^ Number of emergency department patients in last 4 h. ^c^ Number of opioids ever prescribed to patient during all previous emergency department visits. *SD* standard deviation, *IQR* interquartile range

**Fig. 1:**
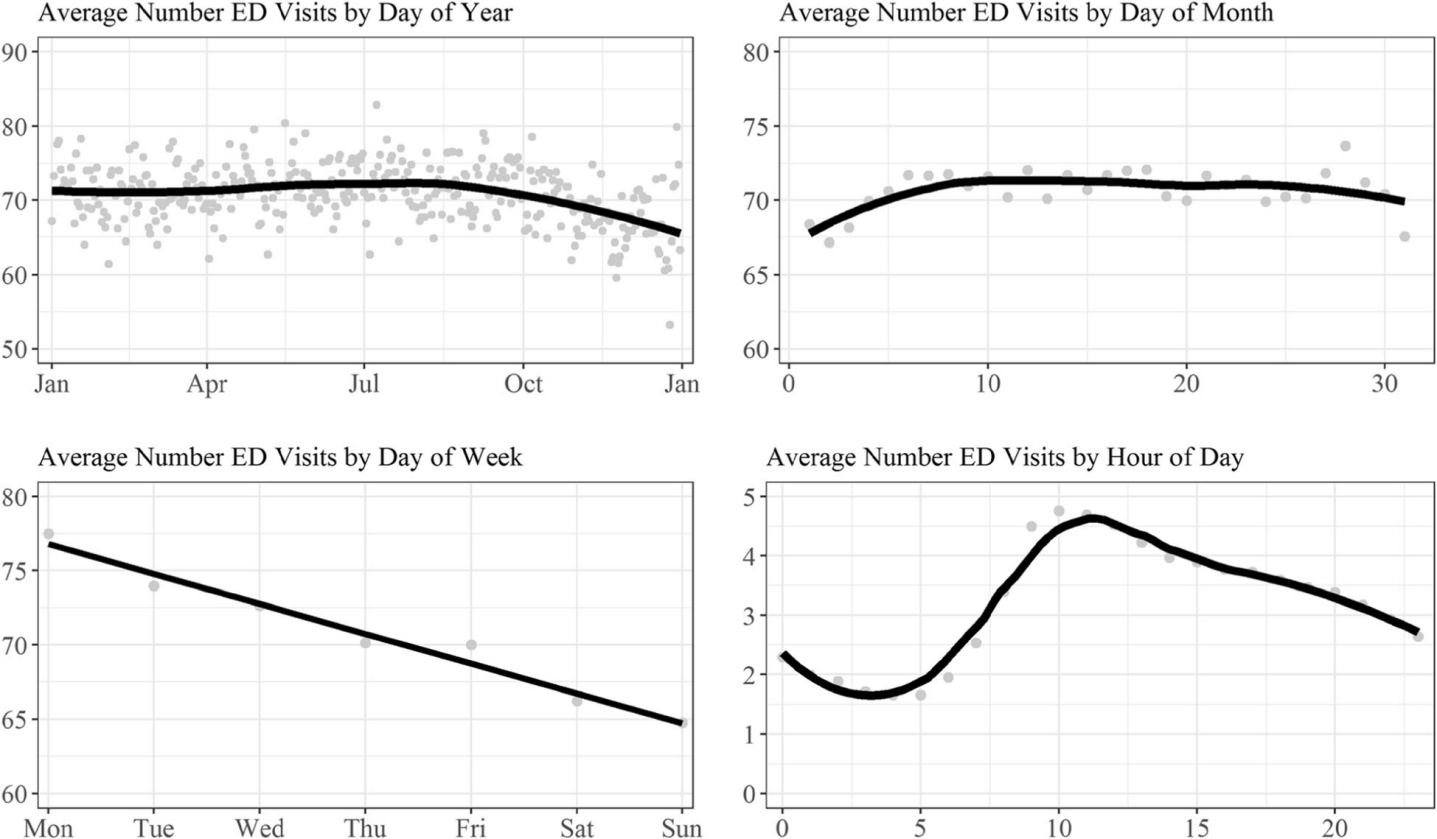
Number of Emergency Department (ED) Visits by Unit of Time. Note: Sample includes all electronic medical records from hospital’s emergency department (*n* = 180,829 events; 63,513 unique individuals). Points indicate individual estimates; lines indicate locally estimated scatterplot smoothing (LOESS) curves

Figure 1 illustrates patterns of ED visits over time. There are relatively minimal annual and monthly trends in ED crowding, with ED crowding appearing smallest towards the end of the year and at the beginning of the month. However, the ED was noticeably less crowded towards the end of the week and during late hours of the day. These results suggest that conditioning for the time of a patients’ visit may be necessary for determining the potential underlying association between ED crowding and opioid prescribing.

Table 2 provides three mixed models for opioid prescribing during a given ED visit, with Model 2 controlling for ICD-9 diagnosis and Model 3 using fixed effects to control for all time-invariant heterogeneity between patients as well as ICD-9 diagnosis. Parameter estimates are reported in average marginal effects, with a 0.01 unit increase in a given parameter representing a one-percentage point increase in the probability that the provider will prescribe opioids. Parameter estimates for the original models are reported in log odds and are available in Additional file 1.

**Table 2:** Logistic Regression Models Predicting Opioid Prescription During Emergency Department Visit

	Model 1^**a**^			Model 2^**b**^			Model 3^**c**^		
	AME		95% CI	AME		95% CI	AME		95% CI
**Contextual Variables**									
ED crowding^d^	−0.002	***	[− 0.002,− 0.001]	-0.001	***	[− 0.002,-0.001]	− 0.002	***	[− 0.005,-0.002]
ED crowding *x* black	0.000		[− 0.001,0.001]	0.000		[−0.001,0.001]	0.000		[−0.003,0.002]
Time^e^									
*6 am-11:59 am*	0.008	*	[0.001,0.015]	−0.005		[−0.01,0.001]	0.018		[0.004,0.030]
*12 pm-5:59 pm*	−0.009	*	[−0.016,-0.001]	− 0.014	*	[− 0.019,-0.010]	− 0.006		[− 0.027,0.017]
*6 pm-11:59 pm*	− 0.021	***	[− 0.028,-0.013]	− 0.020	***	[− 0.023,-0.017]	− 0.026	***	[− 0.033,-0.019]
Weekend	0.022	***	[0.017,0.026]	0.014	***	[0.011,0.016]	0.029	***	[0.025,0.032]
Year	−0.022	***	[− 0.023,-0.021]	− 0.025	***	[− 0.025,-0.024]	− 0.028	***	[− 0.032,-0.026]
Prev. prescribed (#)	0.081	***	[0.080,0.083]	0.063	***	[0.061,0.064]	0.114	***	[0.109,0.118]
**Demographic Variables**									
Age 20–30	−0.012		[− 0.055,0.031]	− 0.006		[− 0.031,0.018]			
Age 30–40	0.007		[−0.036,0.050]	0.011		[−0.013,0.035]			
Age 40–50	0.019		[−0.024,0.062]	0.019		[−0.005,0.044]			
Age 50–60	0.004		[−0.039,0.048]	0.004		[−0.022,0.031]			
Age 60–70	−0.009		[−0.053,0.035]	−0.017		[−0.044,0.010]			
Age 70–80	−0.055	**	[−0.100,-0.010]	−0.063	**	[−0.093,-0.033]			
Age 80–90	−0.094	***	[−0.140,-0.049]	−0.119	***	[−0.140,-0.099]			
Age 90+	−0.128	***	[−0.179,-0.077]	−0.152	***	[−0.182,-0.123]			
Race^f^									
*Black*	−0.033	***	[−0.045,-0.02]	−0.018	**	[−0.029,-0.007]			
*Latino*	0.024	***	[0.010,0.039]	0.000		[−0.008,0.008]			
*Asian*	−0.049	**	[−0.083,-0.014]	−0.040	*	[−0.058,-0.022]			
*Other*	0.007		[−0.014,0.027]	−0.004		[−0.017,0.008]			
Marital Status^g^									
*Married*	0.021	***	[0.015,0.027]	0.014	***	[0.012,0.017]			
*Divorced*	0.002		[−0.008,0.011]	0.006		[−0.002,0.014]			
*Widowed*	0.016	*	[0.004,0.028]	0.012	*	[0.004,0.019]			
*Separated*	−0.002		[−0.015,0.011]	0.001		[−0.006,0.009]			
Sex									
*Female*	0.001		[−0.008,0.009]	0.003		[−0.004,0.010]			
*Female x Black*	−0.011	*	[−0.022,-0.001]	−0.007		[−0.013,0.001]			
*Female x Latino*	−0.021	*	[−0.040,-0.002]	−0.004		[−0.018,0.009]			
*Female x Asian*	−0.022		[−0.068,0.024]	−0.005		[−0.009,0.001]			
*Female x Other*	−0.023		[−0.050,0.005]	−0.020		[−0.044,0.003]			

Notes: * *p* < 0.05; ** *p* < 0.01; *** *p* < 0.001. Parameter estimates reported in average marginal effects. Full models include polynomial terms and interaction effects between prev. Prescribed (#) and year. Sample includes all EMR from hospital ED (*n* = 180,829 events; 63,513 unique individuals). Years of analysis = 2008–2014. ^a^ Includes within-person random effects. ^b^ Includes within-person random effects and ICD9 diagnosis. ^c^ Includes within-person fixed effects and ICD9 diagnosis. ^d^ Number of ED patients in last 4 h. ^e^ Reference time = 12 am −5:59 am. ^f^ Reference race = White. ^g^ Reference marital status = Unmarried. *ED* emergency department, *AME* average marginal effects, *CI* confidence interval

Parameter estimates for contextual variables are similar across models. ED crowding was associated with a reduced probability for providers to prescribe opioids (*b* = − 0.002 in Model 3; 95% CI = − 0.005 to − 0.002), suggesting that providers are less likely to prescribe opioids during crowded hours. The coefficient for ED crowding implies that patients who visited the ED during mean rates of crowding (13.92 patients) had a 2.78% reduced probability to receive an opioid prescription than patients who visited the ED while it was empty. There was no significant association for the interaction between ED crowding and black patients across models (*b* = 0.000 in Model 3), indicating no significant difference in how ED crowding affected provider prescribing towards black and other patients. The parameter estimate for events from 6 pm-11:59 pm (*b* = − 0.026 in Model 3) is negative, indicating that providers were less likely to prescribe opioids in the evening compared to overnight (12 am to 6 am). In contrast, the estimate for events on the weekend (*b* = 0.029 in Model 3) is positive, indicating that providers were more likely to prescribe opioids on weekends. The coefficient for year is negative (*b* = − 0.028 in Model 3), implying that providers have become less likely to prescribe opioids over this sample period. Moreover, the models imply that providers were substantially more likely to prescribe opioids to patients that had been previously prescribed opioids at the ED (*b* = 0.114 in Model 3; 95% CI = 0.109 to 0.118).

Parameter estimates for demographic variables are unavailable for Model 3 due to their time-invariance but are mostly consistent between Model 1 and Model 2. In these models, individuals’ probability to receive opioids decreases after age 70 (*b* = − 0.063 in Model 2). Patients that are married (*b* = 0.014) and widowed (*b* = 0.012) have an increased probability to be prescribed opioids compared to the unmarried. Although black and Latino females were less likely to be prescribed opioids in Model 1 (b = − 0.011 and b = − 0.021, respectively), there are no differences after controlling for ICD-9 diagnosis. Without controlling for ICD-9 diagnosis (as illustrated in Model 1), providers have a lower probability to prescribe opioids to patients that are black (*b* = − 0.033) and Asian (*b* = − 0.049), and a higher probability to prescribe opioids to individuals who are Latino (*b* = 0.024). However, controlling for diagnosis in Model 2 resulted in negative parameter estimates for black and Asian patients but no difference for Latinos relative to white patients. Together, these changes indicate a negative association between non-white race and receiving opioid analgesics once we control for racial differences in health conditions.

As a supplemental analysis, we also examined prescription of non-opioid pain medications. Results for non-opioids were consistent with results for opioids: prescription of pain medications decreases during periods of ED crowding (see full results in Additional file 2). However, reversing the effect for opioids, black patients were more likely to be prescribed non-opioids than white patients.^2^ We also include a histogram of previous opioid prescriptions (Additional file 3) for reference.

## Discussion

Prior research has identified multiple factors that can bias provider prescribing of opioids; however, evidence is often mixed. We theorized that previous discrepant findings might result from overlooked contextual and demographic influences on prescribing behavior. Therefore, we modeled how multiple institutional and cultural constraints work in tandem to influence provider prescription of opioids with data from an emergency department’s electronic medical records. Overall, our results provide explanations to previous inconsistencies, and suggest new factors that might influence opioid prescribing.

Most importantly, we found that doctors are less likely to prescribe opioids during ED crowding, and we found no race interaction with ED crowding, implying that ED crowding reduces provider opioid prescription rates similarly across all races. In part, reduced prescriptions during times of crowding may reflect providers’ reduced time for all activities during such periods. We may also attribute this finding to risk-aversion: providers are aware that opioid analgesics have severe negative side effects and they err on the side of caution when their ability to evaluate patient needs is constrained [4, 25]. Our risk-aversion interpretation is consistent with our findings that providers are less likely to prescribe opioids to greater risk demographics (older patients), lower-information patients (those with fewer previous opioid prescriptions), and during periods of time where the harms of opioid analgesics are more salient (more recent years of analysis). Conflicting findings likely arise from differences in study samples. For example, Neprash and Barnett [23] focused their analysis on a sample of pre-scheduled primary care office visits of patients that had a history with a given doctor. Compared to providers in EDs, providers in primary care offices have a greater amount of information about their patients and a much stronger motivation towards maintaining those relationships. Perhaps in such contexts, providers’ aversion to displeasing their clientele surpasses their aversion to over-prescribing opioid analgesics. Likewise, Lara-Millán’s [15] ethnographic study of ED crowding and opioid prescribing for black patients was drawn from a hospital that appeared to be under-resourced. Perhaps underfunding further influences providers away from prioritizing quality care.

This study also produced three additional findings. First, even net of diagnoses and between-patient heterogeneity, we found a strong preference for doctors to prescribe opioids to patients who had previously received opioids from the ED. Emergency departments often have repeat patient visitors, and it is possible that some of the prescribing patterns have to do with giving patients” what they want.” It may be that some patients present to the ED often, with the direct purpose of obtaining pain medications. Some providers may prescribe to these patients quickly in order to get on to the next patient. This type of prescribing may be a future area of research, as it accounts for a significant area of prescribing bias. Second, we found weekly and daily patterns of ED crowding. Providers were less likely to prescribe opioids after 6 pm and also on the weekends. It may be that the timing of ED visits is a function of convenience for the patient. That is, patients may delay coming to the ED after 6 pm even though they are in pain for the ease of ED presentation. Alternatively, many patients may lack evening and weekend transportation, so future analyses should attempt to measure these additional sources of variation in ED visits and crowding over time. Even though we controlled for patient ICD-9 diagnoses, these classifications likely fail to wholly capture patients’ needs for analgesic treatment. Finally, we found that providers are less likely to prescribe opioid analgesics to racial minorities relative to white patients, especially Asians, even after taking diagnostic differences into account. Perhaps most perniciously, black patients were less likely to be prescribed opioids and more likely to be prescribed non-opioids compared to white patients. This is consistent with suggestions that black patients face systemic biases in obtaining treatment for pain [33]. That said, we have little ability to distinguish whether these treatment decisions were influenced by provider biases or sociocultural differences in patients’ presentations and preferences. In either case, these disparities imply that racial minorities are undertreated for pain.

Most other findings were consistent with the literature. Individuals with valued familial ties, such as those who are married or widowed, were significantly more likely to be prescribed opioid analgesics. Higher-risk age groups, such as seniors, were significantly less likely to receive opioids. Patients who visited the ED in later years were less likely to receive opioid analgesics, indicating a cautioning effect of the opioid crisis. In addition, we found no evidence for female or female-race interaction effects on opioid analgesic prescribing after taking diagnoses into account. This null finding does not necessarily contradict studies which find a female effect on opioid prescribing; however, it suggests that gender biases in opioid treatment may only occur under particular diagnostic contexts. Future research should attempt to identify what types of contexts may produce stronger gender biases.

This study had several limitations. First, the study only examined prescribing habits during the years 2008–2014; factors associated with prescribing bias may have changed since this period. Importantly, the rich information contained in these data suggest that findings from them will be informative despite their date. Moreover, many sources of provider bias that we had observed have been noted in the literature for decades. Likewise, we conducted supplemental analyses of prescriber biases with data only from 2008 and data only from 2014 and we found substantively identical results to those reported in the manuscript at both time points. Taken together, this evidence suggests that the factors we identified to be associated with prescriber bias during 2008–2014 are likely to be highly stable over time and potentially still present today. Second, the study only examined prescribing habits in one hospital ED. The ED of our analysis is unlikely to be representative of all other EDs in the United States; our sample is better funded than most EDs and the demographic composition of our ED’s patients over-represents women, African Americans, and unmarried individuals. Third, the analysis was conducted on secondary data rather than on experimental results, thereby limiting the analysis’ ability to differentiate factors that may cause provider bias from factors that are simply correlated with provider bias. Fourth, our measurement of ED crowding is limited in that it does not directly measure the number of patients in an ED waiting room or the amount of time that each patient was seen by a given provider. We assume that EDs are more crowded when increasing numbers of patients visited the ED, net of time of day and day of the week. However, daily variations in the time that some patients take to treat and the number of providers available in the ED almost certainly weaken the accuracy of our measurement of ED crowding. Future research might explore whether alternative measures of crowding change the findings presented here. Lastly, much of our discussion assumes that factors which reduce providers’ likelihood to prescribe opioids leads to undertreatment; however, data limitations (and the inherent ambiguity of opioid diagnosis) prevent us from determining whether patients in these conditions were truly undertreated. Factors that we found to have a downward association with opioid pain treatment may have actually served to prevent overtreatment.

## Conclusions

We found that within-patient differences in the receipt of an opioid prescription during a visit to the ED was negatively associated with visiting the ED at higher rates of crowding, later times of day, earlier days of the week, at later years, and with having received few opioid prescriptions during previous ED visits. Across patients, we found that providers were more likely to prescribe opioids to patients who were middle-aged, white, and married. These results reaffirm previous findings that institutional constraints and racial demographics can significantly bias how providers prescribe opioid analgesics and suggest new paths that may bias providers in analgesic treatment. Given the current state of the opioid epidemic in America, our findings that providers tend to err towards more cautious treatment for pain during constrained diagnostic situations may seem encouraging. However, it is critical that we do not forget the costs of undertreatment as well. To identify the consequences of undertreatment, future research should estimate the direct effects of provider biases in opioid analgesic prescribing on patients’ long-term health. It is essential that the field continues to work towards identifying institutional policies that can lead to consistent and appropriate treatment for pain.

## Supplementary Information

**Additional file 1.** Full logistic regression results for Models 1–3 in log odds.

**Additional file 2.** Logistic regression results for non-opioid prescriptions.

**Additional file 3.** Histogram for previous opioid prescriptions.

## Data Availability

The dataset generated and analyzed during the current study is not publicly available to protect the privacy of participants but it is available from the corresponding author on reasonable request.

## Abbreviations

EMR: Electronic Medical Records
ED: Emergency Department
ICD-9: International Classification of Diseases

## Footnotes

1 We also examined prescription of 3 different non-opioid pain medications in a supplemental analysis (see Additional file 2). This analysis will further investigate the influence of ED crowding on physicians’ prescribing behaviors.

2 ED crowding was associated with a reduced probability for providers to prescribe non-opioid pain medications (*b* = − 0.002 in Model 3 of Additional file 2). Reversing the effect for opioids, black patients were more likely to be prescribed non-opioids than white patients (*b =* 0.025 in Model 2). This is consistent with the possibility that black patients are undertreated for pain. In contrast to opioids, non-opioid prescriptions were more likely to be prescribed overnight (12 am–6 am), in more recent years, and to younger patients. Full results are in Additional file 2.
