# Supplementary material for: Provider Bias in prescribing opioid analgesics: a study of electronic medical Records at a Hospital Emergency Department
Source: BMC Public Health. 2021 Aug 6;21:1518. doi: 10.1186/s12889-021-11551-9 (PMC8344207; doi:10.1186/s12889-021-11551-9)
Supplement: Supplementary file 1 — Additional file 1. Full logistic regression results for Models 1–3 in log odds. [file 12889_2021_11551_MOESM1_ESM.docx]

|  | **Appendix Table 1. Logistic Regression Models for Opioid Prescription During ED Visit** | | | | | | |  |  |  |  |  |  |  |  |
| --- | --- | --- | --- | --- | --- | --- | --- | --- | --- | --- | --- | --- | --- | --- | --- |
|  |  | **Model 1^a^** | | **Model 2^b^** | | **Model 3^c^** | |  |  |  |  |  |  |  |  |
|  | **Contextual Variables** |  |  |  |  |  |  |  |  |  |  |  |  |  |  |
|  | ED crowding^d^ | -0.010 | *** | -0.009 | *** | - 0.014 | *** |  |  |  |  |  |  |  |  |
|  | ED crowding *x*Black | -0.000 |  | -0.001 |  | 0.000 |  |  |  |  |  |  |  |  |  |
|  | 6am - 12pm | 0.047 | * | -0.035 |  | - 0.034 |  |  |  |  |  |  |  |  |  |
|  | 12pm - 6pm | -0.051 | * | -0.098 | *** | - 0.157 | *** |  |  |  |  |  |  |  |  |
|  | 6pm - 12am | -0.119 | *** | -0.138 | *** | - 0.197 | *** |  |  |  |  |  |  |  |  |
|  | Weekend | 0.127 | *** | 0.095 | *** | 0.136 | *** |  |  |  |  |  |  |  |  |
|  | Year | 0.005 |  | -0.061 | *** | - 0.169 | *** |  |  |  |  |  |  |  |  |
|  | **Patient Variables** |  |  |  |  |  |  |  |  |  |  |  |  |  |  |
|  | Prev. prescribed (#) | 0.816 | *** | 0.728 | *** | 0.697 | *** |  |  |  |  |  |  |  |  |
|  | Prev. prescribed (#)^2^ | -0.026 | *** | -0.023 | *** | - 0.020 | *** |  |  |  |  |  |  |  |  |
|  | Prev. prescribed (#) *x Year* | -0.085 | *** | -0.070 | *** | - 0.079 | *** |  |  |  |  |  |  |  |  |
|  | Prev. prescribed (#)^2^ *x Year* | 0.003 | *** | 0.002 | *** | 0.003 | *** |  |  |  |  |  |  |  |  |
|  | Age 20-30 | -0.069 |  | -0.043 |  |  |  |  |  |  |  |  |  |  |  |
|  | Age 30-40 | 0.040 |  | 0.074 |  |  |  |  |  |  |  |  |  |  |  |
|  | Age 40-50 | 0.110 |  | 0.134 |  |  |  |  |  |  |  |  |  |  |  |
|  | Age 50-60 | 0.024 |  | 0.031 |  |  |  |  |  |  |  |  |  |  |  |
|  | Age 60-70 | -0.051 |  | -0.118 |  |  |  |  |  |  |  |  |  |  |  |
|  | Age 70-80 | -0.319 | * | -0.435 | ** |  |  |  |  |  |  |  |  |  |  |
|  | Age 80-90 | -0.546 | *** | -0.824 | *** |  |  |  |  |  |  |  |  |  |  |
|  | Age 90+ | -0.742 | *** | -1.050 | *** |  |  |  |  |  |  |  |  |  |  |
|  | Race^h^ |  |  |  |  |  |  |  |  |  |  |  |  |  |  |
|  | *Black* | -0.189 | *** | -0.122 | ** |  |  |  |  |  |  |  |  |  |  |
|  | *Latino* | 0.141 | *** | 0.002 |  |  |  |  |  |  |  |  |  |  |  |
|  | *Asian* | -0.281 | ** | -0.276 | * |  |  |  |  |  |  |  |  |  |  |
|  | *Other* | 0.038 |  | -0.029 |  |  |  |  |  |  |  |  |  |  |  |
|  | Marital Status^i^ |  |  |  |  |  |  |  |  |  |  |  |  |  |  |
|  | *Married* | 0.122 | *** | 0.098 | *** |  |  |  |  |  |  |  |  |  |  |
|  | *Divorced* | 0.011 |  | 0.042 |  |  |  |  |  |  |  |  |  |  |  |
|  | *Widowed* | 0.092 | * | 0.080 | * |  |  |  |  |  |  |  |  |  |  |
|  | *Separated* | -0.012 |  | 0.009 |  |  |  |  |  |  |  |  |  |  |  |
|  | Sex |  |  |  |  |  |  |  |  |  |  |  |  |  |  |
|  | *Female* | 0.004 |  | 0.021 |  |  |  |  |  |  |  |  |  |  |  |
|  | *Female x Black* | -0.129 |  | -0.141 |  |  |  |  |  |  |  |  |  |  |  |
|  | *Female x Latino* | -0.063 | * | -0.045 |  |  |  |  |  |  |  |  |  |  |  |
|  | *Female x Asian* | -0.120 | * | -0.032 |  |  |  |  |  |  |  |  |  |  |  |
|  | *Female x Other* | -0.131 |  | -0.031 |  |  |  |  |  |  |  |  |  |  |  |
|  | **Intercept** | -1.230 | *** | -2.123 | *** |  |  |  |  |  |  |  |  |  |  |
|  | Notes: * p < 0.05; ** p < 0.01; *** p < 0.001. Parameter estimates reported in log odds. Data from all electronic medical records (EMR) from hospital's emergency department (ED). Years of analysis = 2008-2014. n = 180,829 events; 63,513 unique individuals. a) Includes within-person random effects. b) Includes within-person random effects and ICD-9 diagnosis c) Includes within-person fixed effects and ICD9 diagnosis. d) Number of ED patients in last 4 hours. e) 0 = 5am. f) 0 = Monday. g) 0 = 2008. h) Reference = White. i) Reference = Unmarried. | | | | | | |  |  |  |  |  |  |  |  |
|  |  |  |  |  |  |  |  |  |  |  |  |  |  |  |  |
|  |  |  |  |  |  |  |  |  |  |  |  |  |  |  |  |
|  |  |  |  |  |  |  |  |  |  |  |  |  |  |  |  |
|  |  |  |  |  |  |  |  |  |  |  |  |  |  |  |  |
|  |  |  |  |  |  |  |  |  |  |  |  |  |  |  |  |
|  |  |  |  |  |  |  |  |  |  |  |  |  |  |  |  |
|  |  |  |  |  |  |  |  |  |  |  |  |  |  |  |  |
|  |  |  |  |  |  |  |  |  |  |  |  |  |  |  |  |
|  |  |  |  |  |  |  |  |  |  |  |  |  |  |  |  |
|  |  |  |  |  |  |  |  |  |  |  |  |  |  |  |  |
|  |  |  |  |  |  |  |  |  |  |  |  |  |  |  |  |
|  |  |  |  |  |  |  |  |  |  |  |  |  |  |  |  |
|  |  |  |  |  |  |  |  |  |  |  |  |  |  |  |  |
|  |  |  |  |  |  |  |  |  |  |  |  |  |  |  |  |
|  |  |  |  |  |  |  |  |  |  |  |  |  |  |  |  |
|  |  |  |  |  |  |  |  |  |  |  |  |  |  |  |  |
|  |  |  |  |  |  |  |  |  |  |  |  |  |  |  |  |
|  |  |  |  |  |  |  |  |  |  |  |  |  |  |  |  |
|  |  |  |  |  |  |  |  |  |  |  |  |  |  |  |  |
|  |  |  |  |  |  |  |  |  |  |  |  |  |  |  |  |
|  |  |  |  |  |  |  |  |  |  |  |  |  |  |  |  |
|  |  |  |  |  |  |  |  |  |  |  |  |  |  |  |  |
|  |  |  |  |  |  |  |  |  |  |  |  |  |  |  |  |
|  |  |  |  |  |  |  |  |  |  |  |  |  |  |  |  |
|  |  |  |  |  |  |  |  |  |  |  |  |  |  |  |  |
|  |  |  |  |  |  |  |  |  |  |  |  |  |  |  |  |
|  |  |  |  |  |  |  |  |  |  |  |  |  |  |  |  |
|  |  |  |  |  |  |  |  |  |  |  |  |  |  |  |  |
|  |  |  |  |  |  |  |  |  |  |  |  |  |  |  |  |
