# Supplementary material for: Provider Bias in prescribing opioid analgesics: a study of electronic medical Records at a Hospital Emergency Department
Source: BMC Public Health. 2021 Aug 6;21:1518. doi: 10.1186/s12889-021-11551-9 (PMC8344207; doi:10.1186/s12889-021-11551-9)
Supplement: Supplementary file 2 — Additional file 2. Logistic regression results for non-opioid prescriptions. [file 12889_2021_11551_MOESM2_ESM.docx]

| **Appendix Table 2. - Logistic Regression Models for Non-Opioid Prescription During Emergency Department Visit** | | | | | | | | | |
| --- | --- | --- | --- | --- | --- | --- | --- | --- | --- |
|  |  |  |  |  |  |  |  |  |  |
|  | **Model 1^a^** | | | **Model 2^b^** | | | **Model 3^c^** | | |
|  | **AME** | | **95% CI** | **AME** | | **95% CI** | **AME** | | **95% CI** |
| **Contextual Variables** |  |  |  |  |  |  |  |  |  |
| ED crowding^d^ | -0.001 | ** | [-0.001,-0.000] | -0.001 | *** | [-0.002,-0.000] | -0.002 | *** | [-0.004,-0.001] |
| ED crowding *x*Black | -0.001 | * | [-0.001,-0.000] | -0.001 |  | [-0.001,0.000] | 0.000 |  | [-0.002,0.001] |
| 6am - 12pm | 0.023 | *** | [0.016,0.030] | 0.011 | ** | [0.003,0.019] | 0.012 | * | [0.003,0.021] |
| 12pm - 6pm | 0.007 |  | [-0.001,0.015] | 0.000 |  | [-0.009,0.008] | -0.004 |  | [-0.016,0.009] |
| 6pm - 12am | 0.000 |  | [-0.008,0.008] | -0.003 |  | [-0.012,0.006] | -0.005 |  | [-0.018,0.008] |
| Weekend | 0.003 |  | [-0.002,0.008] | 0.002 |  | [-0.004,0.007] | 0.006 |  | [-0.002,0.013] |
| Year | 0.020 | *** | [0.018,0.021] | 0.015 | *** | [0.014,0.016] | 0.005 | *** | [0.002,0.008] |
| Prev. prescribed (#) | 0.029 | *** | [0.028,0.03] | 0.024 | *** | [0.023,0.024] | 0.023 | *** | [0.021,0.026] |
| **Demographic Variables** |  |  |  |  |  |  |  |  |  |
| Age 20-30 | 0.102 | *** | [0.043,0.162] | 0.099 | *** | [0.060,0.137] |  |  |  |
| Age 30-40 | 0.119 | *** | [0.059,0.179] | 0.110 | *** | [0.070,0.149] |  |  |  |
| Age 40-50 | 0.136 | *** | [0.076,0.196] | 0.115 | *** | [0.074,0.155] |  |  |  |
| Age 50-60 | 0.130 | *** | [0.070,0.191] | 0.105 | *** | [0.064,0.146] |  |  |  |
| Age 60-70 | 0.121 | *** | [0.061,0.181] | 0.091 | ** | [0.050,0.131] |  |  |  |
| Age 70-80 | 0.101 | ** | [0.040,0.162] | 0.064 | * | [0.023,0.106] |  |  |  |
| Age 80-90 | 0.082 | ** | [0.021,0.144] | 0.030 |  | [-0.011,0.072] |  |  |  |
| Age 90+ | 0.058 |  | [-0.007,0.122] | 0.007 |  | [-0.039,0.053] |  |  |  |
| Race^e^ |  |  |  |  |  |  |  |  |  |
| *Black* | 0.030 | *** | [0.018,0.043] | 0.025 | *** | [0.014,0.037] |  |  |  |
| *Latino* | 0.021 | ** | [0.006,0.036] | 0.008 |  | [-0.002,0.017] |  |  |  |
| *Asian* | -0.015 |  | [-0.050,0.019] | -0.016 |  | [-0.039,0.007] |  |  |  |
| *Other* | 0.017 |  | [-0.003,0.037] | 0.010 |  | [-0.010,0.029] |  |  |  |
| Marital Status^f^ |  |  |  |  |  |  |  |  |  |
| *Married* | 0.000 |  | [-0.006,0.006] | 0.001 | *** | [-0.002,0.003] |  |  |  |
| *Divorced* | -0.001 |  | [-0.010,0.008] | -0.002 |  | [-0.009,0.004] |  |  |  |
| *Widowed* | 0.006 |  | [-0.006,0.018] | 0.000 | * | [-0.009,0.010] |  |  |  |
| *Separated* | 0.003 |  | [-0.010,0.016] | 0.001 |  | [-0.008,0.010] |  |  |  |
| Sex |  |  |  |  |  |  |  |  |  |
| *Female* | -0.009 | * | [-0.018,-0.001] | 0.002 |  | [-0.005,0.008] |  |  |  |
| *Female x Black* | -0.004 |  | [-0.015,0.007] | -0.003 |  | [-0.011,0.005] |  |  |  |
| *Female x Latino* | -0.027 |  | [-0.047,-0.007] | -0.003 |  | [-0.013,0.007] |  |  |  |
| *Female x Asian* | -0.019 | ** | [-0.066,0.028] | -0.009 |  | [-0.029,0.012] |  |  |  |
| *Female x Other* | -0.029 | * | [-0.057,-0.001] | -0.015 |  | [-0.030,-0.001] |  |  |  |
| Notes: * p < 0.05; ** p < 0.01; *** p < 0.001. Sample includes all EMR from hospital ED (n = 180,829 events; 63,513 unique individuals). Years of analysis = 2008-2014. a) Includes within-person random effects. b) Includes within-person random effects and ICD9 diagnosis. c) Includes within-person fixed effects and ICD9 diagnosis. d) Number of ED patients in last 4 hours. e) Reference race = White. f) Reference marital status = Unmarried. AME, average marginal effects; ED, emergency department; CI, confidence interval. | | | | | | | | | |
|  |  |  |  |  |  |  |  |  |  |
|  |  |  |  |  |  |  |  |  |  |
|  |  |  |  |  |  |  |  |  |  |
