# Supplementary figures and images for: Provider Bias in prescribing opioid analgesics: a study of electronic medical Records at a Hospital Emergency Department
Source: BMC Public Health. 2021 Aug 6;21:1518. doi: 10.1186/s12889-021-11551-9 (PMC8344207; doi:10.1186/s12889-021-11551-9)

# Histogram of Previous Opioid Prescriptions

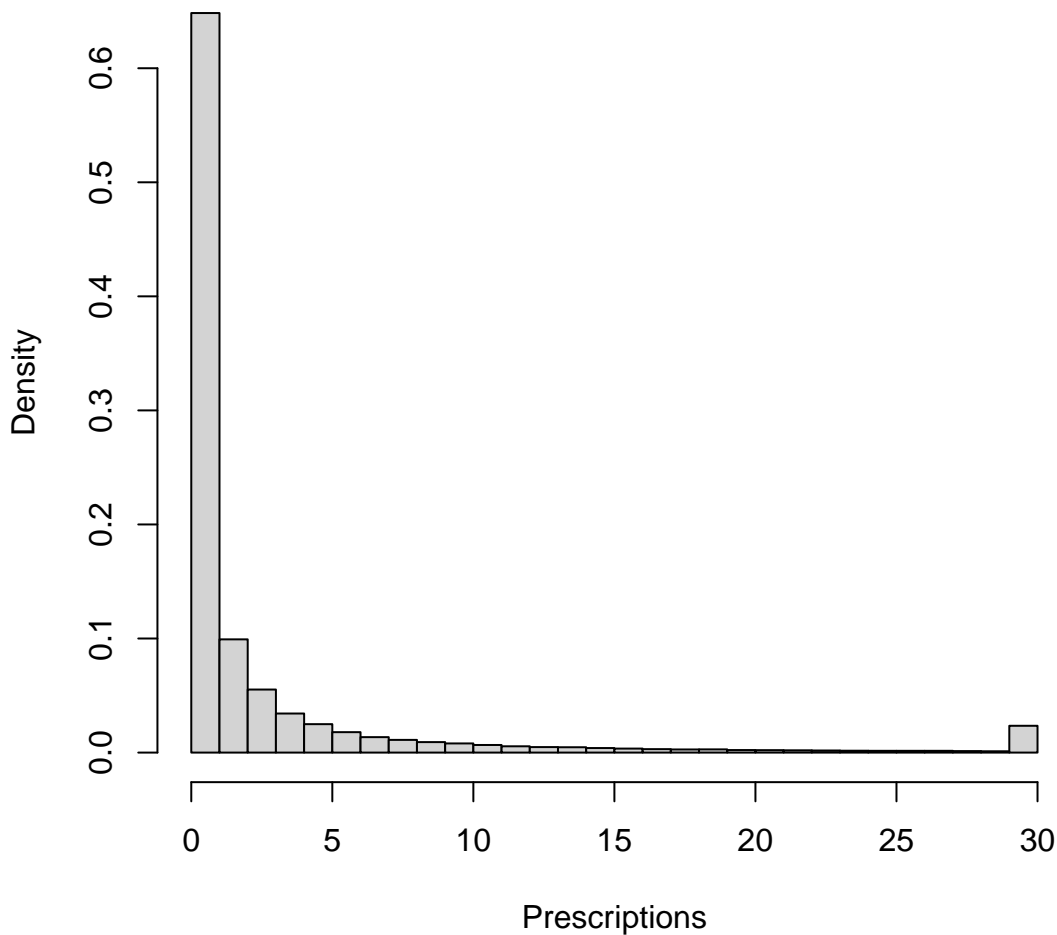

Supplement: Supplementary file 3 — Additional file 3. Histogram for previous opioid prescriptions. [file 12889_2021_11551_MOESM3_ESM.pdf]
